# Comparison of Brain Natriuretic Peptide Levels to Simultaneously Obtained Right Heart Hemodynamics in Stable Outpatients with Pulmonary Arterial Hypertension

**DOI:** 10.3390/diseases6020033

**Published:** 2018-05-01

**Authors:** Scott A. Helgeson, J. Saadi Imam, John E. Moss, David O. Hodge, Charles D. Burger

**Affiliations:** 1 Department of Internal Medicine, Mayo Clinic, Jacksonville, FL 32224, USA; helgeson.scott@mayo.edu; 2 Department of Pulmonary & Critical Care Medicine, The University of Texas Medical Branch, Galveston, TX 77555, USA; jsimam@utmb.edu; 3 Department of Critical Care Medicine, Mayo Clinic, Jacksonville, FL 32224, USA; moss.john@mayo.edu; 4 Department of Biomedical Statistics and Informatics, Mayo Clinic, Jacksonville, FL 32224, USA; hodge@mayo.edu; 5 Department of Pulmonary and Critical Care Medicine, Mayo Clinic, Jacksonville, FL 32224, USA

**Keywords:** pulmonary arterial hypertension, brain natriuretic peptide, biomarkers, right heart catheterization, transthoracic echocardiogram

## Abstract

Pulmonary arterial hypertension (PAH) is a progressive disease that requires validated biomarkers of disease severity. While PAH is defined hemodynamically by right heart catheterization (RHC), brain natriuretic peptide (BNP) is recommended by guidelines to assess disease status. Retrospectively collected data in 138 group 1 PAH patients were examined for the correlation of BNP levels to simultaneously obtained right heart catheterization (RHC). Patients were mostly Caucasian women, with functional class III symptoms, mean BNP of 406 ± 443 pg/mL, and an average right atrial pressure (RAP) of 9.9 ± 5.7 mm Hg and mean pulmonary artery pressure (mPAP) of 47.3 ± 14.7 mm Hg. Significant correlation was demonstrated between BNP and RAP (*p* = 0.021) and mPAP (*p* = 0.003). Additional correlation was seen with right heart size on echocardiography: right atrial (RAE; *p* = 0.04) and right ventricular enlargement (*p* = 0.03). An increased BNP level was an independent predictor of mortality (*p* < 0.0001), along with RAP (*p* = 0.039) and RAE (*p* = 0.018). Simultaneous collection of BNP at the time of RHC confirmed the correlation of BNP with right heart hemodynamics. The current results reinforce the use of BNP level as a continuous variable to assess disease severity in group 1 PAH.

## 1. Introduction

Pulmonary arterial hypertension (PAH) is a progressive disease caused by endothelial proliferation, inflammation, and remodeling of the pulmonary vasculature. These changes in pulmonary vasculature result in narrowing or obliteration of the vessel lumen, thereby increasing resistance to pulmonary arterial blood flow. The right heart hypertrophies to generate higher upstream pressure to overcome the elevated pulmonary vascular resistance (PVR). In the event of undertreatment or treatment failure, the right ventricle (RV) enlarges to the point of failure, leading to cor pulmonale and death. Five-year survival without PAH therapy is approximately 60%; however, appropriate therapy may double life expectancy. Monitoring disease progression and treatment response is therefore critical to appropriate management of this patient population [1,2,3]. The assessment of disease status and treatment response assessment is often challenging as there is no ideal biomarker in this regard. Since PAH is hemodynamically defined, patients often must undergo a right heart catheterization (RHC), an invasive and costly procedure, to restage the disease or monitor treatment response.

Nonetheless, other disease severity markers are promoted in published guidelines to assess disease status during follow-ups, such as brain natriuretic peptide (BNP) or N-terminal pro-BNP or the six-minute walk test (6MWT). Indeed, serum levels of BNP correlate with PAH prognosis and have been incorporated into guideline recommendations to characterize patients as lower risk (normal levels) or higher risk (BNP > 180 pg/mL) [4,5,6]. In addition, BNP levels have demonstrated a correlation with the World Health Organization functional class (WHO-FC) and hemodynamic parameters established by RHC in patients with PAH [7,8]. In patients with RV overload, BNP levels have been shown to correlate with mean pulmonary artery pressure (mPAP) and PVR [8]. Decreased survival has also been seen in PAH patients with BNP levels greater than 180 pg/mL [4,6].

Limitations of the studies that have focused primarily on the BNP include sample size, cohort inclusion of unstable patients, and missing disease severity markers (e.g., WHO-FC, 6MWT, etc.). Larger studies such as the Registry to Evaluate Early and Long-Term PAH Management (REVEAL) include a plethora of disease severity markers; however, hemodynamic assessment is only required at diagnosis. If a subsequent RHC is performed, the hemodynamic values are only included if obtained within the last year of the current assessment. Lastly, the hemodynamic values that are incorporated into the REVEAL risk score are only pertinent if extremely abnormal, for example, right atrial pressure (RAP) > 20 mm Hg or PVR > 32 Wood units.

In our view, there was potential value in assessing BNP levels that were obtained simultaneously to the hemodynamic parameters from RHC to more precisely determine the correlation and impact on outcome. Additional value was sought from WHO-FC, 6MWT, and transthoracic echocardiographic (TTE) assessment of right heart size and function.

The primary aim of our study was to examine the correlation between the largest cohort of simultaneously obtained BNP level and hemodynamics by RHC in group 1 PAH outpatients. The study also focused on the correlation of BNP as a continuous variable, rather than an arbitrary cutoff level, compared to mortality in PAH patients. Secondary aims included comparison of BNP level to WHO-FC, 6MWT results (six-minute walk distance [6MWD] and heart rate recovery), and right heart size as assessed by TTE.

## 2. Materials and Methods

We reviewed and analyzed retrospectively collected data from a subspecialty pulmonary hypertension (PH) practice at a single center (Mayo Clinic, Jacksonville, FL, USA) between 20 July 2004 and 31 August 2015. This study’s protocol was approved by the institutional review board (IRB). An electronic medical record review identified diagnostic group 1 PAH patients diagnosed by RHC, with initial hemodynamic measurements of mPAP ≥ 25 mm Hg, PVR > 3 Wood units, and pulmonary capillary wedge pressure (PCWP) ≤ 15 mm Hg, and excluded non-group 1 patients by standard guideline recommendations [9,10]. A PCWP > 15 mm Hg was permitted if the left ventricular end-diastolic pressure was ≤15 mm Hg. Only patients with a BNP measured simultaneously with their initial RHC and an initial TTE performed within one week of the BNP were included in this study. The study was a retrospective analysis on data routinely obtained and recorded in our PH center clinical database. Exclusion criteria included impaired renal function, defined as a serum creatinine level greater than 1.3 mg/dL and/or creatinine clearance less than 50 mL/min calculated from the Cockcroft–Gault equation, and impaired left ventricular function, defined as a left ventricular ejection fraction less than 40%. Patients were also excluded if they were admitted to the hospital with acute right or left heart decompensation. All patients were older than 18 years old.

The WHO-FC was determined for each patient using a standardized patient classification system [11]. Plasma BNP concentrations were collected in ethylenediamine tetra-acetic acid tubes and assayed on an Alere Triage^®^ system using a fluorescence immunoassay on the same day as the RHC and within 7 days of the TTE. The 6MWT was performed using a standardized protocol following the American Thoracic Society statement 2002 [12]. The distance walked was recorded as 6MWD. The maximum heart rate during the walk was recorded as the peak heart rate. Additionally, a one-minute post-walk heart rate was obtained. Those two heart rates were used to calculate the heart rate recovery (HRR). A threshold of less than 16 beats per minute in HRR has been shown previously to predict a poor outcome [13].

Two-dimensional TTE data was obtained through standard transthoracic windows using a 2.5-MHz transducer. The parameters used for this study included right atrial enlargement (RAE), right ventricular enlargement (RVE), and right ventricular dysfunction (RVD). The echocardiographic data of the right atrium and the right ventricle was described semiquantitatively as normal (1), mildly (2), moderately (4), or severely (6) dilated/hypertrophied, or noted to have abnormal function. The results were obtained by the initial interpretation of the TTE by an echocardiography board-certified cardiologist that routinely reads for the PH Center; therefore, an over-read was not performed. Data collected from the RHC included RAP, mPAP, PCWP, and cardiac output (CO). The CO was calculated by the Fick method using indirect calorimetry by metabolic cart (MGC Diagnostics). The cardiac index (CI) was extrapolated from the calculated CO and the patient’s body surface area. The PVR was calculated by dividing mPAP minus PCWP by CO.

The data were presented as the mean ± SD. A statistical software package, SAS version 9.4, was utilized for analysis by a biostatistician. Log transformation was used to normalize the distribution of BNP levels. The overall distribution of BNP was skewed given the large range, so a log transformation was used to make the variable closer to normal. All results were adjusted using this log transformation. Correlations of the log of BNP and each of the other parameters of interest were obtained using Pearson’s correlation coefficients. Overall mortality was estimated using the Kaplan–Meier method. The univariate and multivariate relationship of each of the parameters to mortality was evaluated using Cox proportional hazards models. Due to constraints with the incident size, multiple multivariate analyses consisting of WHO FC, 6MWD, HRR, TTE parameters, RHC hemodynamic parameters, and BNP as a continuous variable had to be performed. One large multivariate analysis could not be performed with all these variables, so separate analyses for each group of variables was performed. The groups were WHO FC, BNP, 6MWT parameters, right heart size by TTE, and RHC hemodynamic parameters. Univariate analysis for mortality was performed on BNP levels of 76 pg/mL, 180 pg/mL, and 240 pg/mL. The rationale was as follows: 76 pg/mL was the upper limit of normal for our laboratory, 180 pg/mL was determined to be a published cutoff level for PAH patients, and 240 pg/mL was determined to be the mean value of our cohort [6]. The combinations were (1) BNP and RAE > 4, (2) BNP and RAP > 9 mm Hg, (3) BNP and HRR, and (4) BNP, RAE > 4, RAP > 9 mm Hg, and HRR. These pairings were chosen to represent different components of an evaluation of a patient with PAH, including laboratory results, 6MWT, TTE, and RHC. A *p*-value less than 0.05 was considered statistically significant.

## 3. Results

A total of 163 patients with PAH had simultaneous BNP with RHC and TTE performed within the predefined time window. Twenty-one patients were excluded based on elevated creatinine of greater than 1.3 mg/dL and four patients based on decreased left ventricular ejection fraction of less than 40%. Ultimately, 138 patients with PAH met all the inclusion and exclusion criteria. Baseline patient characteristics, WHO-FC, BNP level, 6MWT results, and TTE parameters are summarized in Table 1. Patients were mostly Caucasian women with WHO-FC III symptoms. The distribution of the PAH classification was as follows: idiopathic (*n* = 54), connective tissue disease-related (*n* = 54), portal hypertension-related (*n* = 16), drug- or toxin-induced (*n* = 6), congenital heart disease-related (*n* = 5), heritable PAH (*n* = 2), and human immunodeficiency virus infection (*n* = 1).

The mean BNP level for all patients was 405.9 ± 443.1 pg/mL (median was 239). The natural log of the BNP levels showed significant positive correlations with RAP, mPAP, and PVR, and negative correlations with both CO and CI calculated from the values obtained during the simultaneous RHC (Table 2). No correlation was seen with PCWP. In comparison to right heart size by TTE, BNP positively correlated with RAE, RVE, and RVD (Table 2). The TTE and 6MWT were performed within 3.1 ± 2.2 days and 4.3 ± 12.3 days, respectively, from obtaining the BNP levels. The 6MWT yielded strong negative correlations to BNP level and 6MWD. The HRR also correlated negatively with 6MWD (Table 2). There was a statistically significant correlation between BNP and WHO-FC (Table 2).

**Figure 1:**
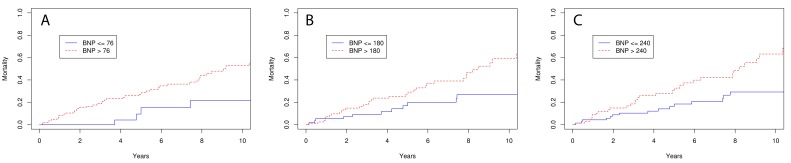
Kaplan–Meier mortality curves for (**A**) BNP cutoff of 76 pg/mL, (**B**) BNP cutoff of 180 pg/mL, and (**C**) BNP cutoff of 240 pg/mL.

Overall mortality for our cohort was 46% at ten years. There was a significant relationship with all three BNP levels (76 pg/mL, 180 pg/mL, and 240 pg/mL) and mortality (Table 3). Kaplan–Meier mortality curves were not statistically different between each level of BNP measured at 5 and 10 years (Figure 1), while BNP levels in the higher range had a stronger correlation. The correlation was most significant when BNP was analyzed as a continuous variable. Other variables that demonstrated a relationship to mortality were RAP, RAE, HRR, and 6MWD (Table 3). A multivariate analysis was performed on these variables and demonstrated that BNP (*p* < 0.001), HRR (*p* < 0.02), and RAE (*p* < 0.016) were significantly related to mortality (Table 4).

## 4. Discussion

The primary aim of our study was to examine the relationship between simultaneously obtained BNP level and hemodynamics by RHC in group 1 PAH outpatients. In this study, we demonstrated that BNP levels significantly correlated with all of the examined hemodynamic variables except PCWP. Our secondary aims to compare BNP to WHO-FC, 6MWD, HRR, right heart size, and mortality demonstrated highly significant correlations between BNP and WHO-FC as well as 6MWD and HRR. The BNP level correlated to a lesser extent with right heart size based on TTE measurements. We also discovered that any abnormally elevated BNP level correlated with poorer outcome. Indeed, any elevation in BNP was more significant than any specific cutoff, a result that differs somewhat from previously published results [2]. Additionally, a significant univariate relationship to mortality existed only for RAP, RAE, and RVD. While the multivariate analysis was limited as described in the methods, RAE was the only variable that showed an increase in mortality as enlargement increased.

By comparison, plasma BNP levels have previously been shown to correlate with various hemodynamic parameters of the right heart and have a prognostic value in patients with PAH [6,7,8]. These studies were performed with small cohorts and prior to significant medication-related advances in PAH management. This present study is the largest and most current study performed evaluating BNP and hemodynamic variables obtained simultaneously in stable outpatients. This study also incorporated patients that had both a new and prior diagnosis of PAH. We show that this correlation is stronger with hemodynamics than with right heart size.

The TTE has been used to assess disease severity in patients with PAH without significant evidence to support its benefit. The assumption has been that a TTE may show long-term effects of increased pressures in the heart and will be able to identify the high-risk features of estimated RAP > 15 mm Hg, tricuspid regurgitation ≥ moderate, and the presence of a pericardial effusion, values that all correlate with disease severity and mortality [8,14,15,16]. Nonetheless, there has been only one study comparing BNP and TTE variables to mortality in group 3 pulmonary hypertension patients with idiopathic pulmonary fibrosis [17]. The study included 131 adults with pulmonary hypertension caused by idiopathic pulmonary fibrosis and showed that BNP level, but not any TTE variables, was an independent predictor of survival [17]. Our results showed a significant correlation between BNP and not only the size of the heart chambers, but also with hemodynamic dysfunction of the RV, and to the best of our knowledge, this is the first study showing that BNP may be used reliably as a surrogate for TTE-acquired data in stable patients with PAH. The correlation demonstrated in this study may permit contemporaneous disease assessment with a low-cost, readily available biomarker.

An additional interesting result from the current study is that any elevation in BNP level above normal is significant, rather than levels over a specific cutoff. For example, the first study reporting a specific cutoff used a level of ≥180 pg/mL [6]. They showed that survival was much worse for patients with pulmonary hypertension and right heart dysfunction. Our study confirms those results but also demonstrates that survival is more greatly affected for PAH patients with any BNP level in excess of the upper limit of normal (i.e., ≥76 pg/mL). It should be noted the mean BNP in the current study was higher than previously reported. There was also a statistical trend that a higher BNP level was associated with an increased mortality. This finding that increasing BNP levels correlate with increased mortality has very important clinical significance. In the REVEAL trial, the designated BNP levels used for prognostication were ≤50 pg/mL, indicating a favorable one-year survival, and a level of >180 pg/mL as unfavorable [4]. Levels between those values were not found to have any predictive value [4]. In contrast, the results in the current cohort suggest that any elevation in BNP above the normal range can have a significant impact on mortality and should be of concern.

Other means of assessing PAH severity such as WHO-FC, 6MWD, and HRR have previously been utilized. The strongest correlation with any variable in our study was with 6MWD. Our study supports the findings of Leuchte et al., who showed that 6MWD has a strong negative correlation with BNP [9]. HRR is a strong prognostic measure for patients with PAH, but a correlation with BNP has never been shown [13]. An odds ratio of 28 for BNP > 100 pg/mL and HRR < 16 bpm was shown, but in our study there is a very strong correlation between BNP and poor HRR. The fact that these two variables have a strong correlation with BNP strongly suggests that as BNP increases, the WHO FC should also increase.

This study provides supportive evidence of a more prominent role for BNP levels in the monitoring of PAH patients; however, there are significant limitations. While the BNP level was obtained at the same time as the RHC for each individual patient, the TTE and 6MWT were performed the week prior. Cohort bias exists as this data was obtained from a single referral center for PAH patients. In addition, our patient population consisted of both newly diagnosed patients and also patients with a prior diagnosis of PAH, although there did not appear to be any clinically significant differences in demographics or PAH severity. The effect of PAH treatment was not examined.

In conclusion, our data from the largest cohort of simultaneously obtained RHC and BNP reinforced the correlation of BNP with right heart hemodynamic variables and right heart size. Most significantly, any elevation in BNP was associated with worse survival; therefore, it may serve as an indicator of poorer outcomes. As such, any elevation in BNP in group 1 PAH may be clinically significant. The results reinforce the utilization of BNP to evaluate prognostic status, but are additive to current risk scores in that any elevation represents a clinical concern. In addition, as BNP correlated with right heart enlargement and RV dysfunction as expected, regular assessment of BNP with intermittent utilization of TTE seems reasonable and aligns with published guidelines [9]. The result suggests that BNP may be used as a surrogate for some echocardiographic data in PAH patients. Nonetheless, the results would require multicenter, prospective validation before acceptance as true in all circumstances.

## Figures and Tables

**Table 1:** Clinical characteristics of the study cohort.

Variables	All Patients (*n* = 138)	BNP < 240 (*n* = 68)	BNP ≥ 240 (*n* = 70)	*p*-Value for BNP Groups
Age (years)	59.2 ± 13.6	59.4 ± 13.3	59 ± 13.9	0.869
Sex female)	104 (75.4%)	47 (69.1%)	57 (81.4%)	0.115
Race (*n*)				0.513
Caucasian	114 (82.6%)	56 (82.3%)	58 (82.9%)	
African American	15 (10.9%)	9 (13.2%)	6 (8.6%)	
Latino	5 (3.6%)	1 (1.5%)	4 (5.7%)	
Asian	2 (1.4%)	1 (1.5%)	1 (1.4%)	
Native American	1 (0.7%)	0 (0%)	1 (1.4%	
Caribbean	1 (0.7%)	1 (1.5%)	0 (0%)	
Body mass index (kg/m^2^)	27.9 ± 8.4	29.2 ± 10.4	26.8 ± 5.7	0.098
Creatinine clearance (mL/min)	84.3 ± 41.8	90.8 ± 48.9	78 ± 32.7	0.071
WHO functional class (n)				0.007
I	15 (10.97%)	10 (14.7%)	5 (7.1%)	
II	25 (18.1%)	18 (26.5%)	7 (10%)	
III	70 (50.7%)	32 (47. 1%)	38 (54.3%)	
IV	28 (20.3%)	8 (11.8%)	20 (28.67%)	
Brain natriuretic peptide (BNP) (pg/mL)	406 ± 443	99.4 ± 75.4	703.9 ± 448.9	<0.0001
Hemodynamic parameters				
RAP (mm Hg)	9.9 ± 5.7	8.7 ± 5	11.1 ± 6.2	0.021
mPAP (mm Hg)	47.3 ± 14.7	43.1 ± 14.8	51.3 ± 13.4	0.001
PCWP (mm Hg)	12.3 ± 5.4	13.1 ± 3.8	11.1 ± 2.3	0.138
CO (L/min)	4.7 ± 1.9	5.2 ± 2.1	4.2 ± 1.4	0.001
CI (L/min/m^2^)	2.5 ± 0.9	2.7 ± 0.9	2.3 ± 0.7	0.004
PVR (dynes)	681.8 ± 426	541.8 ± 361	826.4 ± 442.2	0.0001
Right heart size by TTE (grade)				
RAE	3.2 ± 2.5	2.8 ± 2.5	3.6 ± 2.4	0.051
RVE	3.4 ± 2.3	2.8 ± 2.2	4 ± 2	0.003
RVD	3 ± 2.3	2.5 ± 2.3	3.5 ± 2.2	0.013
Six-minute walk test				
Distance walked (m)	319.7 ± 119.5	388.8 ± 95.9	249.6 ± 98.8	<0.0001
Heart rate recovery (bpm)	30.1 ± 13.1	37.2 ± 12.2	23.3 ± 10	<0.0001
Heart rate recovery (%)	28.4 ± 10.3	33.9 ± 9	23.2 ± 8	<0.0001
Medication use (*n*)				0.075
Diuretics	49 (35.5%)	17 (25%)	32 (45.7%)	
Calcium channel blockers	25 (18.1%)	10 (14.7%)	15 (21.4%)	
Oral PAH medication	46 (33.3%)	22 (32.4%)	24 (34.3%)	
Inhaled prostacyclin	12 (8.7%)	6 (8.8%)	6 (8.6%)	
Intravenous prostacyclin	11 (7.9%)	6 (8.8%)	5 (7.1%)	

World Health Organization (WHO); right atrial pressure (RAP); mean pulmonary artery pressure (mPAP); pulmonary capillary wedge pressure (PCWP); cardiac output (CO); cardiac index (CI); pulmonary vascular resistance (PVR); transthoracic echocardiogram (TTE); grade is semiquantitatively estimated with 0 = normal, 2 = mild, 4 = moderate, and 6 = severe; right atrial enlargement (RAE); right ventricular enlargement (RVE); right ventricular dysfunction (RVD); pulmonary arterial hypertension (PAH). Results are presented as mean ± SD or *n*. Categorical data was analyzed using a Fisher’s exact test and continuous data was analyzed using a nonparametric Wilcoxson test. Categorical data will not always add up to 100% because of rounding.

**Table 2:** Correlation coefficients between brain natriuretic peptide and all variables.

Variable	r	*p*-Value
Hemodynamics		
RAP	0.202	0.021
mPAP	0.253	0.003
CO	−0.325	0.0002
CI	−0.277	0.002
PCWP	−0.207	0.76
PVR	0.305	0.0006
Right heart size by TTE		
RAE	0.181	0.04
RVE	0.186	0.03
RVD	0.151	0.08
Six-minute walk test		
Heart rate recovery: absolute	−0.654	<0.0001
Heart rate recovery: percentage	−0.700	<0.0001
Distance walked	−0.752	<0.0001
WHO-FC		
Functional class	0.257	0.002

Right atrial pressure (RAP); mean pulmonary artery pressure (mPAP); cardiac output (CO); cardiac index (CI); pulmonary capillary wedge pressure (PCWP); pulmonary vascular resistance (PVR); transthoracic echocardiogram (TTE); right atrial enlargement (RAE); right ventricular enlargement (RVE); right ventricular dysfunction (RVD); World Health Organization functional class (WHO-FC).

**Table 3:** Univariate analysis predictors of mortality in PAH patients.

Variable	Hazard Ratio	95% CI	*p*-Value
Brain natriuretic peptide			
lg BNP: continuous	1.698	1.301–2.215	<0.0001
BNP > 76 pg/mL	3.674	1.317–10.250	0.013
BNP >180 pg/mL	2.540	1.287–5.012	0.007
BNP >240 pg/mL	2.401	1.305–4.417	0.005
Hemodynamics			
RAP	1.055	1.003–1.110	0.039
mPAP	1.013	0.992–1.034	0.227
Cardiac index	0.750	0.522–1.076	0.118
Right heart size by TTE			
RVE	0.923	0.811–1.050	0.224
RVD	0.953	0.838–1.084	0.466
RAE	0.857	0.754–0.974	0.018
WHO-FC			
Functional class	1.425	0.984–2.064	0.061
Six minute walk test			
Heart rate recovery–absolute	0.936	0.909–0.964	<0.0001
Distance walked	0.992	0.990–0.995	<0.0001

Pulmonary arterial hypertension (PAH); Brain natriuretic peptide (BNP); log transformation of BNP (lg BNP); right atrial pressure (RAP); mean pulmonary artery pressure (mPAP); transthoracic echocardiogram (TTE); right atrial enlargement (RAE); right ventricular enlargement (RVE); right ventricular dysfunction (RVD); World Health Organization functional class (WHO-FC).

**Table 4:** Multivariate analysis of predictors of mortality in study cohort.

Variable	Hazard Ratio	95% CI	*p*-Value
lg BNP	1.585	1.199–2.095	0.001
RAP	1.047	0.984–1.113	0.15
mPAP	0.988	0.961–1.016	0.39
Cardiac index	0.854	0.579–1.258	0.42
RVE	0.879	0.658–1.173	0.379
RVD	1.207	0.916–1.59	0.181
RAE	0.802	0.671–0.96	0.016
WHO-FC	1.303	0.895–1.898	0.168
Heart rate recovery: absolute	0.95	0.910–0.992	0.02

Log transformation of brain natriuretic peptide (lg BNP); right atrial pressure (RAP); mean pulmonary artery pressure (mPAP); right ventricular enlargement (RVE); right ventricular dysfunction (RVD); right atrial enlargement (RAE); World Health Organization functional class (WHO-FC).

## References

[1] Austin C., Burger C., Kane G., Safford R., Blackshear J., Ung R., Shapiro B. (2017). High-risk echocardiographic features predict mortality in pulmonary arterial hypertension. Am. Heart J..

[2] Benza R.L., Gomberg-Maitland M., Miller D.P., Frost A., Frantz R.P., Foreman A.J., McGoon M.D. (2012). The REVEAL Registry Risk Score Calculator in Patients Newly Diagnosed With Pulmonary Arterial Hypertension. Chest.

[3] Benza R.L., Miller D.P., Barst R.J., Badesch D.B., Frost A.E., McGoon M.D. (2012). An Evaluation of Long-term Survival from Time of Diagnosis in Pulmonary Arterial Hypertension from the REVEAL Registry. Chest.

[4] Benza R.L., Miller D.P., Gomberg-Maitland M., Frantz R.P., Foreman A.J., Coffey C.S., McGoon M.D. (2010). Predicting survival in pulmonary arterial hypertension: Insights from the Registry to Evaluate Early and Long-Term Pulmonary Arterial Hypertension Disease Management (REVEAL). Circulation.

[5] Galie N., Humbert M., Vachiery J.L., Gibbs S., Lang I., Torbicki A., Hoeper M. (2015). 2015 ESC/ERS Guidelines for the diagnosis and treatment of pulmonary hypertension: The Joint Task Force for the Diagnosis and Treatment of Pulmonary Hypertension of the European Society of Cardiology (ESC) and the European Respiratory Society (ERS): Endorsed by: Association for European Paediatric and Congenital Cardiology (AEPC), International Society for Heart and Lung Transplantation (ISHLT). Eur. Respir. J..

[6] Galiè N., Manes A., Negro L., Palazzini M., Bacchi-Reggiani M.L., Branzi A. (2009). A meta-analysis of randomized controlled trials in pulmonary arterial hypertension. Eur. Heart J..

[7] Hunt S.A., Abraham W.T., Chin M.H., Feldman A.M., Francis G.S., Ganiats T.G., Yancy C.W. (2009). 2009 Focused Update Incorporated Into the ACC/AHA 2005 Guidelines for the Diagnosis and Management of Heart Failure in Adults. J. Am. Coll. Cardiol..

[8] ATS Committee on Proficiency Standards for Clinical Pulmonary Function Laboratories (2002). ATS statement: Guidelines for the six-minute walk test. Am. J. Respir. Crit. Care Med..

[9] Leuchte H.H., Holzapfel M., Baumgartner R.A., Ding I., Neurohr C., Vogeser M., Behr J. (2004). Clinical significance of brain natriuretic peptide in primary pulmonary hypertension. J. Am. Coll. Cardiol..

[10] McLaughlin V.V., Archer S.L., Badesch D.B., Barst R.J., Farber H.W., Lindner J.R., Varga J. (2009). ACCF/AHA 2009 Expert Consensus Document on Pulmonary Hypertension. A Report of the American College of Cardiology Foundation Task Force on Expert Consensus Documents and the American Heart Association Developed in Collaboration With the American College of Chest Physicians; American Thoracic Society, Inc.; and the Pulmonary Hypertension Association. J. Am. Coll. Cardiol..

[11] Minai O.A., Gudavalli R., Mummadi S., Liu X., McCarthy K., Dweik R.A. (2012). Heart rate recovery predicts clinical worsening in patients with pulmonary arterial hypertension. Am. J. Respir. Crit. Care Med..

[12] Miyamoto S., Nagaya N., Satoh T., Kyotani S., Sakamaki F., Fujita M., Miyatake K. (2000). Clinical correlates and prognostic significance of six-minute walk test in patients with primary pulmonary hypertension. Comparison with cardiopulmonary exercise testing. Am. J. Respir. Crit. Care Med..

[13] Nagaya N., Nishikimi T., Okano Y., Uematsu M., Satoh T., Kyotani S., Kangawa K. (1998). Plasma Brain Natriuretic Peptide Levels Increase in Proportion to the Extent of Right Ventricular Dysfunction in Pulmonary Hypertension. J. Am. Coll. Cardiol..

[14] Nagaya N., Nishikimi T., Uematsu M., Satoh T., Kyotani S., Sakamaki F., Kangawa K. (2000). Plasma brain natriuretic peptide as a prognostic indicator in patients with primary pulmonary hypertension. Circulation.

[15] Simonneau G., Gatzoulis M.A., Adatia I., Celermajer D., Denton C., Ghofrani A., Souza R. (2013). Updated clinical classification of pulmonary hypertension. J. Am. Coll. Cardiol..

[16] Wiese S., Breyer T., Dragu A., Wakili R., Burkard T., Schmidt-Schweda S., Holubarsch C.J. (2000). Gene expression of brain natriuretic peptide in isolated atrial and ventricular human myocardium: Influence of angiotensin II and diastolic fiber length. Circulation.

[17] Song J.W., Song J.K., Kim D.S. (2009). Echocardiography and brain natriuretic peptide as prognostic indicators in idiopathic pulmonary fibrosis. Respir. Med..

